# Variability and performance of radiologic stricture parameters in Crohn's disease: a systematic review and meta-analysis

**DOI:** 10.1016/j.eclinm.2025.103541

**Published:** 2025-10-08

**Authors:** Arianna Dal Buono, Francesco Faita, Sarah Bencardino, Giacomo Maiucci, Alberto Barchi, Alessandro Armuzzi, Dominik Bettenworth, Silvio Danese, Mariangela Allocca

**Affiliations:** a IBD Center, Department of Gastroenterology, IRCCS Humanitas Research Hospital, Via Manzoni 56, 20089 Rozzano, Milan, Italy; b Department of Biomedical Sciences, Humanitas University, Via Rita Levi Montalcini 4, 20072 Pieve Emanuele, Milan, Italy; c Institute of Clinical Physiology, Italian National Research Council, Pisa, Italy; d Gastroenterology and Endoscopy, IRCCS Ospedale San Raffaele and University Vita-Salute San Raffaele, Milan, Italy; e Medical Faculty, University of Münster, Münster, Germany; f CED Schwerpunktpraxis, Münster, Germany

**Keywords:** Stricture, Crohn's disease, Intestinal ultrasound, Cross-sectional imaging, Accuracy

## Abstract

**Background:**

Disease-related strictures are a common complication of Crohn's disease (CD). Cross-sectional imaging is widely used for their assessment, but definitions remain variable and non-standardised. This systematic review and meta-analysis aimed to identify commonly used imaging parameters, assess diagnostic performance, and evaluate consistency across studies.

**Methods:**

We conducted a systematic review of ultrasound (US), magnetic resonance imaging (MRI), and computed tomography (CT) studies on CD-associated strictures, searching MEDLINE/PubMed, Embase, and Cochrane to January 1, 2025. We included prospective and retrospective studies of small-bowel CD strictures with surgical histopathology as reference standard. Summary data were extracted from published reports and pooled using a random-effects bivariate meta-analysis, which jointly models sensitivity and specificity while accounting for between-study heterogeneity. Exclusion criteria were pediatric populations, colonic and upper-GI strictures. Main outcomes were stricture definitions and diagnostic performance. The study is registered with PROSPERO, CRD420251032918.

**Findings:**

Of the 9436 articles identified through the search, 30 met eligibility criteria and were included in the analysis, comprising 1866 patients with CD: 5 on US, 7 on CT, 8 on MRI, and the remaining assessed two techniques. Luminal narrowing (LN), bowel wall thickening (BWT), and pre-stenotic dilation (PSD) were the most common descriptors. 4 studies (13%) required all three; the remaining used LN or BWT alone, with PSD often considered optional (20/30 [77%]). Overall, 26.7% (8/30) of studies were judged at high or unclear risk of bias in at least one domain. Pooled sensitivity and specificity for US techniques to detect strictures were 0.88 (95% CI, 0.83–0.91) and 0.86 (95% CI, 0.79–0.91) (*I*^2^ = 0%), respectively. Pooled sensitivity and specificity for MRE were 0.82 (95% CI, 0.69–0.90) and 0.80 (95% CI, 0.44–0.95) (*I*^2^ = 61.2%), and for CTE were 0.83 (95% CI, 0.73–0.90) and 0.77 (95% CI, 0.47–0.93) (*I*^2^ = 58.8%), respectively.

**Interpretation:**

High diagnostic accuracy was observed across imaging modalities, with no statistically significant difference among them, and only a minority of studies at risk of bias unlikely to affect these findings. Heterogeneity existed in cut-offs and parameter combinations used to define strictures. PSD does not appear essential for stricture diagnosis, which could simplify diagnostic protocols. Variations in histological criteria and limited evidence for some modalities may limit generalizability.

**Funding:**

None.

**Research in context**

**Evidence before this study**

Strictures are a frequent complication of Crohn's disease (CD), and cross-sectional imaging modalities such as ultrasound, MRI, and CT are commonly used to detect them. We searched MEDLINE/PubMed, Embase, and the Cochrane Library from inception to January 1, 2025, without language restrictions, including prospective and retrospective studies of small-bowel CD-associated strictures with surgical histopathology as reference standard. Previous studies reported considerable heterogeneity in how strictures are defined and assessed radiologically, with variable use of luminal narrowing, bowel wall thickening, and pre-stenotic dilation. This lack of standardization has limited comparability across studies and reproducibility of imaging findings in clinical practice. Available evidence is largely derived from single-center observational studies of variable quality and risk of bias, and few studies have provided pooled estimates of diagnostic accuracy, which we synthesized using a bivariate meta-analysis where appropriate.

**Added value of this study**

This systematic review and meta-analysis demonstrates substantial inconsistencies in imaging definitions of CD-associated strictures. It identifies the most used parameters—luminal narrowing, bowel wall thickening, and pre-stenotic dilatation—and shows that high diagnostic accuracy can be achieved even when pre-stenotic dilatation is excluded. The study provides pooled diagnostic performance data and offers practical insights into which parameters may be sufficient for reliable stricture detection.

**Implications of all the available evidence**

These findings support the simplification and standardization of imaging criteria for stricture detection in CD. Establishing consensus-based definitions could improve diagnostic consistency, facilitate multicenter trials, and inform future guidelines. Ultimately, this work lays the groundwork for developing validated imaging indices to assess strictures and guide treatment decisions. However, interpretation of these results should consider some limitations: included studies used varying histological criteria, potentially introducing bias, and the limited number of studies for certain modalities, such as CTE, may reduce generalizability.

## Introduction

Crohn's disease (CD) is a chronic inflammatory bowel disease characterised by progressive intestinal damage, with strictures affecting up to 20% of patients within 20 years of diagnosis and up to 50% over the course of their lifetime.^1^, ^2^, ^3^ Strictures and other complications (abscesses, fistulas, perforations) are more common in CD with ileal involvement, particularly in the terminal ileum.^3^^,^^4^ Despite the efficacy of biologics for stricturing CD,^5^, ^6^, ^7^ over 45% of patients require endoscopic or surgical intervention within 10 years of diagnosis.^8^ Detecting strictures remains challenging for gastroenterologists, particularly as luminal narrowing that is not passable with an endoscope may be regarded as a stricture, and although the SES-CD includes a domain for stenosis, reliable assessment remains difficult. Over the years, cross-sectional imaging techniques, including intestinal ultrasonography (IUS), magnetic resonance imaging (MRI), and computed tomography (CT), have become increasingly relevant for diagnosing strictures in CD. Current ECCO-ESGAR guidelines recommend the use of IUS and/or MRI, but do not specify a preferred imaging modality.^9^ IUS, a radiation-free, point-of-care, and cost-effective imaging tool, is becoming more accessible, with fewer technical challenges compared to MRI and CT. However, MRI and CT offer more detailed assessments, particularly for evaluating proximal intestinal loops.^10^ Multiple systematic reviews and meta-analyses have demonstrated the accuracy of these imaging techniques in detecting small bowel strictures in CD, with IUS showing sensitivity and specificity ranging from 80%–100%–63% to 100%, respectively, when using histopathology as the reference standard.^11^, ^12^, ^13^, ^14^, ^15^ However, the results are often heterogeneous, depending on the IUS technique used (B-mode, contrast-enhanced ultrasonography [CEUS], or small intestine contrast ultrasonography [SICUS]).^11^, ^12^, ^13^, ^14^ Diagnostic accuracy for stricture detection is comparable between MRI, CT and IUS.^11^, ^12^, ^13^, ^14^ Despite consensus guidelines for defining intestinal strictures via these imaging modalities,^9^^,^^16^ substantial variability remains in the definitions used across studies, including differences in the criteria for stricture diagnosis. Luminal narrowing and pre-stenotic dilation are commonly used, but their proposed cut-offs lack robust evidence, especially for IUS, with some studies failing to provide clear definitions. With these premises, we conducted an updated systematic review and meta-analysis to (a) highlight the heterogeneity in current definitions of intestinal strictures, (b) summarise and evaluate the diagnostic performance of cross-sectional imaging techniques compared to histopathology from surgical specimens, and (c) provide practical guidance on the use of these imaging modalities in the assessment of CD-associated strictures in various clinical settings, adopting a heuristic approach.

## Methods

### Search strategy and selection criteria

This systematic review was carried out following the guidelines of the Cochrane Handbook^17^ and the reporting recommendations of the Preferred Reporting Items for Systematic Reviews and Meta-Analyses (PRISMA).^18^^,^^19^ The protocol of this systematic review and meta-analysis was registered on PROSPERO (CRD420251032918).

This study is a systematic review and meta-analysis of previously published data and does not involve any new studies with human participants or animals performed by the authors. Therefore, ethical approval and informed consent were not required.

Patients and the public were not involved in the design, conduct, reporting, or dissemination of this systematic review and meta-analysis, as it is a secondary analysis of previously published data.

We performed the systematic literature search for published and unpublished studies on MEDLINE-Pubmed, Embase and Cochrane databases updated from inception to January 1, 2025. The primary search strategy was based using tools implemented in the above-mentioned databases. “Title and abstract” (TiAb for Pubmed or ti,ab,kw for Embase) search was implemented in the search string in order to identify the specific key terms in the title and in the body of the abstract of available studies. MESH terms were either implemented in the search strategy where available together with “AND”, “OR”, “NOT” or “NEXT” Boolean operators. The following keywords were used in the search string: “Crohn disease”, “cross-sectional”, “ultrasonography”, “sonography”, “ultrasound”, “computed tomography”, “tomography”, “ct”, “cte”, “mri”, “magnetic resonance”, “enterography”, “enteroclysis”, “mre”. Only manuscripts in English and studies published in peer-reviewed journals were included. The duplicates were evidenced and removed by non-automated tools and the potential eligibility of articles was assessed firstly by titles and abstracts evaluation independently by SB and GM; the whole reviewing and selection process was performed using Rayyan - AI Powered Tool for Systematic Literature Reviews (http://rayyan.qcri.org). The full text of manuscripts was analysed according to inclusion criteria and final decision for inclusion was made after a detailed review of articles bodies. The evaluation of full texts of all relevant articles was performed by SB, GM, ADB and AB. Disagreements were resolved by discussion between all the authors. The detailed searching strategy is shown in Supplementary Table S1.

Observational prospective and retrospective clinical studies, providing a descriptive evaluation of stricture in patients with CD, with the involvement of the lower GI tract (exclusively small-bowel strictures), using diverse cross-sectional imaging techniques, particularly MRI, CT, MRI-enterography (MRE), CT-enterography (CTE) and IUS (with different ultrasound techniques) were included. Surgical histopathology was chosen as the reference standard due to its high accuracy in confirming the presence of intestinal stricture. While variations exist in detailed histologic assessments (e.g., fibrosis grading), the macroscopic identification of stricture in resected specimens provides a consistent and minimally biased outcome for diagnostic accuracy analyses. Exclusion criteria were: studies providing only an endoscopic qualitative evaluation of the stenosis, studies not providing surgical specimen analysis with histopathologic evaluation of the stricture, studies evaluating exclusively elastography, studies on ulcerative colitis (UC) or indeterminate colitis, studies only reporting stenosis diagnosis without providing descriptive data, studies assessing colonic strictures or upper-GI strictures, non-human studies, case reports, case series with <10 patients, abstracts, letters to editor, editorials, commentaries, narrative and systematic reviews (with or without meta-analysis), studies not providing full-text, non-English studies. Patients under 15 years of age were excluded to ensure population homogeneity, given differences in disease behavior and imaging approaches in pediatric CD.

### Data analysis

The following data were extracted from the retrieved articles: study design, publication year, country, total number of patients, gender, age, IBD diagnosis, cross-sectional imaging modality, reference standard comparator (histopathology), radiologic features for stricture definition (meaningly pre-stenotic dilation, luminal narrowing and bowel wall thickening) as per guidelines minimum criteria for stricture diagnosis.

As regards data for meta-analysis, the following parameters related to stricture detection were extracted: prevalence, sensitivity, specificity, true positive (TP), true negative (TN), false positive (FP) and false negative (FN).

Two review authors (ADB and FF) independently extracted all data using a standardised form. All extracted data were then cross-checked for accuracy by both reviewers, and any discrepancies were resolved through discussion, with the involvement of a third author if necessary.

The risk of bias was assessed using the QUADAS-2 tool for evaluating the quality of non-randomised studies included in meta-analyses.^20^ According to the score, all studies were evaluated by three perspectives: the selection of the study groups, the comparability of groups and the ascertainment of the outcome. In case of disagreements, conflict was resolved by discussion between all the authors. We assessed the certainty of evidence for diagnostic accuracy outcomes using the GRADE approach for tests of diagnosis, as recommended by the Cochrane Handbook.

The studies that reported TP, TN, FP and FN were considered eligible for the meta-analysis. When these values were not available, their estimation was obtained from the extracted specificity, sensitivity and prevalence.

A bivariate model was employed to compute pooled sensitivity and specificity. Furthermore, Diagnostic Odds Ratio (DOR) was assessed as a measure of diagnostic accuracy using a bivariate random effects model. All calculations were performed with 95% confidence intervals (CIs). Bivariate *I*^2^ for the included studies was calculated to quantitatively evaluate heterogeneity among the studies, with an I-squared index exceeding 50% indicates moderate heterogeneity, and above 75% high heterogeneity. Forest plots were generated to summarise sensitivity and specificity of studies. An exploratory leave-one-out sensitivity analysis and subgroup analysis (by risk of bias) was performed to assess the robustness of the pooled estimates.

In the event that a study analysed two or more imaging methods, the specific population was considered for the meta-analysis separately for all analyzable methods. A further meta-analysis was conducted excluding pre-stenotic dilation from the diagnostic definition of the stricture. In the case of US imaging method, a further subgroup meta-analysis was performed considering IUS and SICUS studies separately.

Number Needed to Treat (NTT) (under the hypothesis of 50% prevalence) was derived from pooled sensitivity and specificity in order to compare different imaging technologies overall. More in detail, risk in not treated group (RNTG) and risk in treated group (RTG) were evaluated according to the following equations:

RNTG = prevalence

RTG = RNTG ∗ (1 — PNV)

PNV (predictive negative value) = [(1–prevalence) ∗ specificity]/[(1–prevalence) ∗ specificity + prevalence ∗ (1—sensitivity)]

Finally, NTT was obtained from 1/(RNTG–RTG) and z-test was used to compare NTTs from different meta-analysis.

MetaDisc v. 2.0 software for meta-analysis of diagnostic test accuracy was used.

### Role of the funding source

There was no funding source for this study. All authors had full access to the data in the study. The corresponding author had final responsibility for the decision to submit the manuscript for publication.

## Results

**Fig. 1:**
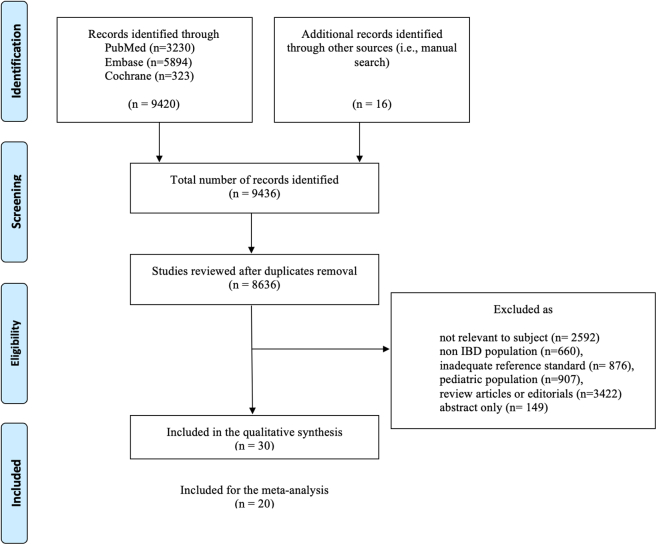
**Preferred reporting items for systematic reviews flow diagram**. PRISMA flow diagram showing study selection: of 9436 records identified, 30 studies (1999–2023) met inclusion criteria, with 8 previously included in Bettenworth et al.

Overall, 9436 articles were retrieved with the search strings used, and after duplicate removal, a total of 8636 papers were assessed by title and abstract evaluation. Ultimately, 142 full texts were assessed for eligibility, and applying the inclusion criteria 30 full-text papers, published between 1999 and 2023, were included in the final analysis.^21^, ^22^, ^23^, ^24^, ^25^, ^26^, ^27^, ^28^, ^29^, ^30^, ^31^, ^32^, ^33^, ^34^, ^35^, ^36^, ^37^, ^38^, ^39^, ^40^, ^41^, ^42^, ^43^, ^44^, ^45^, ^46^, ^47^, ^48^, ^49^, ^50^ Among those, 8 papers retrieved and re-evaluated were already included in the comprehensive systematic analysis by Bettenworth et al.^14^ Fig. 1 illustrates the screening and selection process. The included studies comprised single- and multi-center experiences. The study designs were as follows: 15 of 30 studies were prospective,^21^, ^22^, ^23^^,^^26^, ^27^, ^28^, ^29^, ^30^, ^31^^,^^34^^,^^40^^,^^42^^,^^44^^,^^46^^,^^47^ 14 retrospective studies,^24^^,^^25^^,^^32^^,^^33^^,^^36^, ^37^, ^38^, ^39^^,^^41^^,^^43^^,^^45^^,^^48^^,^^49^^,^^51^ and one comprised both a retrospective and a prospective cohort.^50^ In 4 studies (13%), a control group was included, either with healthy individuals or through comparison with the normal intestinal wall of the same patients.^26^^,^^27^^,^^38^^,^^47^ Inter-observer agreement was assessed in 12 of the 30 included studies (40%) and revealed moderate-to-excellent agreement.^21^^,^^33^^,^^36^, ^37^, ^38^, ^39^^,^^42^^,^^44^^,^^45^^,^^47^^,^^49^^,^^50^ Table 1 provides details of all the studies included.

**Table 1:** Studies included in the qualitative analysis.

Reference	Year	Study design	*N*^a^	Imaging method	Small/large bowel	Evaluation of ref stand
Allocca M et al.^21^	2023	prospective, single-center	17 (16)	IUS	Small and large bowel	Qualitative and semi-quantitative
Servais L et al.^22^	2022	prospective, single-center	17 (10)	IUS, CEUS, MRE	Small and large bowel	Qualitative and semi-quantitative
Viganò L et al.^23^	2019	prospective, single-center	65 (61)	IUS + intra-operatory US, MRE	Small and large bowel	Qualitative and semi-quantitative
Kumar S et al.^25^	2015	retrospective, single-center	42 (8)	IUS, SICUS, MRE	Small bowel	Qualitative and semi-quantitative
Onali S et al.^26^	2012	prospective, single-center	13	IUS, SICUS, CTE	Small and large bowel	Qualitative and semi-quantitative
Pallotta N et al.^27^	2012	prospective, single-center	40	IUS, SICUS	Small bowel	Qualitative and semi-quantitative
Neye H et al.^28^	2010	prospective, single-center	53 (28)	IUS	Small and large bowel	Qualitative
Parente F et al.^29^	2002	prospective, single-center	85 (70)	IUS	Small and large bowel	Qualitative
Gasche C et al. 30	1999	prospective, single-center	33 (22)	IUS	Small and large bowel	Qualitative
Kohn A et al.^31^	1999	prospective, single-center	44 (16)	IUS	Small and large bowel	Qualitative
Dane B et al.^34^	2023	retrospective, single-center	69 (35)	CTE, MRE	Small and large bowel	Qualitative and semi-quantitative
Meng J et al.^36^	2022	retrospective, multi-center	174	CTE	Small and large bowel	Qualitative and semi-quantitative
Duan M et al.^51^	2022	retrospective, single-center	239	CTE	Small and large bowel	Qualitative and semi-quantitative
Hong JT et al.^37^	2022	retrospective, single-center	83	CT, CTE, MRE	Small and large bowel	Qualitative and semi-quantitative
Dane B et al.^32^	2021	retrospective, single-center	16 (10)	dual-CTE	Small and large bowel	Qualitative and semi-quantitative
Li XH et al.^35^	2021	retrospective and prospective cohorts, cross-sectional, single-center	121	CTE	Small and large bowel	Qualitative and semi-quantitative
Stocker D et al.^38^	2021	retrospective, single-center	111^b^	CTE, MRE	Small bowel	Qualitative and semi-quantitative
Saade C et al.^39^	2019	retrospective, single-center	89	CTE	Small and large bowel	Qualitative and semi-quantitative
Pellino G et al.^40^	2016	prospective, single-center	29	CTE/hybrid PET	Small and large bowel	Qualitative and semi-quantitative
Chiorean MV et al.^24^	2007	retrospective, single-center	44	CTE	Small bowel	Qualitative and semi-quantitative
Vogel J et al.^41^	2007	retrospective, single-center	18	CTE	Small bowel	Qualitative and semi-quantitative
Scharitzer M et al.^42^	2023	prospective, single-center	14	MRE/PET	Small bowel	Qualitative and semi-quantitative
Loch FN et al.^49^	2022	retrospective, single-center	67	MRE	Small bowel	Qualitative and semi-quantitative
Foti PV et al.^43^	2021	retrospective, single-center	23	MRE, DWI MRE	Small bowel	Qualitative and semi-quantitative
Fang ZN et al.^44^	2020	retrospective, single-center	41	MRE, magnetization transfer MR	Small bowel	Qualitative and semi-quantitative
Barat M et al.^45^	2019	prospective, single-center	38 (31)	MRE, DWI MRE	Small and large bowel	Qualitative
Pous-Serrano S et al.^46^	2017	prospective, single-center	38 (27)	MRE	Small and large bowel	Qualitative and semi-quantitative
Spinelli A et al.^34^	2014	prospective, single-center	75 (48)	MRE	Small and large bowel	Qualitative
Sinha R et al.^47^	2013	prospective, single-center	49	MRE	Small and large bowel	Qualitative and semi-quantitative
Ha CY et al.^48^	2011	retrospective, single-center	119 (23)	MRE	Small and large bowel	Qualitative

IBD, inflammatory bowel disease; IUS, intestinal ultrasound; MRE, magnetic resonance entero-graphy; CTE, computed tomography entero-graphy; CEUS, contrast-enhanced ultrasonography; PET, positron emission tomography.

a Exclusively IBD patients, total patients included (strictures only).

b Included controls.

Based on the QUADAS-2 checklist, the overall quality of the studies included was rated as good. Only 8 of the 30 (26.7%) studies were considered at risk of bias according to the ratings across various domains (Supplementary Table S2). No study was excluded due to quality concerns.

Regarding patient demographics, a total of 1866 patients with CD were included, approximately half of the affected individuals were male, and in 472 cases (25%), gender was not specified. The age of included patients ranged from 15 to 86 years.

As for the imaging techniques investigated, IUS was the sole imaging modality in 5 studies, CTE (including dual-energy CTE) in 7 studies, MRE in 8 studies (Table 1). In the remaining studies two techniques were addressed simultaneously (i.e., IUS and MRE, IUS and CTE, CTE and MRE, CTE/MRE and PET). Finally, small intestine contrast ultrasonography (SICUS) was assessed in 3 studies,^25^, ^26^, ^27^ contrast-enhanced ultrasonography (CEUS) in one study.^22^

### Definition of stricture and diagnostic accuracy of IUS

**Fig. 2:**
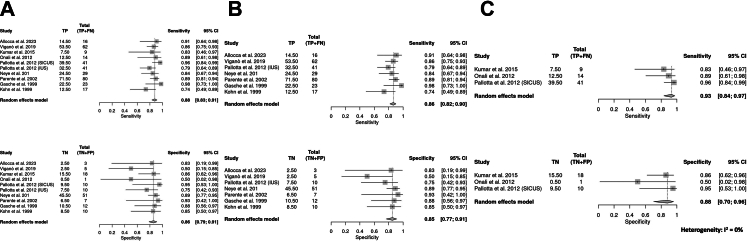
**Pooled diagnostic accuracy of ultrasound techniques**. (A) Overall pooled sensitivity (0.88, 95% CI, 0.83–0.91) and specificity (0.86, 95% CI, 0.79–0.91) with DOR of 39.87; (B and C) subgroup analyses of IUS and SICUS showing comparable sensitivities and specificities.

8/10 studies provided a definition for stricture^21^^,^^25^, ^26^, ^27^, ^28^, ^29^, ^30^, ^31^ (Table 2). In the included studies, the three primary criteria for defining stricture were luminal narrowing (less than 10 mm, <50% as compared to normal adjacent loops, or unspecified), increased bowel wall thickening (typically greater than 3 mm), and/or pre-stenotic dilation (≥25 mm, ≥30 mm, or unspecified). Table 2 illustrates the core criteria for the definition of stricture adopted by the included studies. In detail, luminal narrowing was considered a defining criterion for stricture in 6 out of 10 studies,^21^^,^^26^, ^27^, ^28^, ^29^ with two specifying a narrowing of <10 mm.^26^^,^^27^ Increased bowel wall thickening was regarded as a defining criterion for stricture in 6 out of 10 studies (60%),^21^^,^^25^^,^^28^, ^29^, ^30^, ^31^ with three different cut-offs (>3 mm, >4 mm, >5 mm); in the remaining studies, bowel wall thickening was consistently assessed but not explicitly included as a defining parameter. Pre-stenotic dilation upstream of the stenosis was considered a defining criterion for stricture in only 3 out of 10 studies (30%),^27^^,^^29^^,^^31^ assessed but deemed optional in 6 out of 10 (60%),^21^^,^^22^^,^^25^^,^^26^^,^^28^^,^^30^ and not evaluated in one study.^23^ Nine studies (90%) were suitable for meta-analysis,^21^^,^^23^^,^^25^, ^26^, ^27^, ^28^, ^29^, ^30^, ^31^ one study was excluded for insufficient and non-comparable data.^22^ Pooled sensitivity and specificity for all US techniques (IUS and SICUS) were 0.88 (95% CI, 0.83–0.91) and 0.86 (95% CI, 0.79–0.91), respectively (Fig. 2a). DOR was found to be 39.87 (95% CI, 19.68–80.76). The pooled data were homogeneous (*I*^2^ = 0%). Subgroup analysis assessing the diagnostic accuracy of IUS and SICUS separately showed similar results, with sensitivities of 86% (82–90%) and 93% (84–97%), and specificities of 85% (77–92%) and 88% (70–96%), respectively (Fig. 2b and c).

**Table 2:** Definition of stricture and parameters assessed by technique.

	Reference	Year	Required parameters for stricture diagnosis/Definition	Criterion for luminal narrowing (LN)	Criterion for bowel wall thickening (BWT)	Criterion for pre-stenotic dilation (PSD)
IUS	Allocca M et al.^21^	2023	LN + BWT; PSD optional	Yes	Yes, >3 mm	Evaluated, but not part of definition
	Servais L et al.^22^	2022		Not assessed	Evaluated, but not part of definition	Evaluated, but not part of definition
	Viganò L et al.^23^	2019		Not assessed	Evaluated, but not part of definition	Not assessed
	Kumar S et al.^25^	2015	BWT	Not assessed	Yes, >3 mm	Evaluated, but not part of definition
	Onali S et al.^26^	2012	LN (<10 mm); PSD optional	Yes, <10 mm	Evaluated, but not part of definition	Evaluated (>25 mm), but not part of definition
	Pallotta N et al.^27^	2012	LN (<10 mm); PSD optional	Yes, <10 mm	Evaluated, but not part of definition	Yes, >25 mm
	Neye H et al.^28^	2010	LN + BWT (>3 mm); PSD optional	Yes	Yes, >3 mm	Evaluated but not part of the definition
	Parente F et al.^29^	2002	LN + BWT + PSD	Yes	Yes, ≥4 mm	Yes
	Gasche C et al.^30^	1999	LN + BWT (>3 mm) + wall pattern disrupted; PSD optional	Yes	Yes, >3 mm	Evaluated but not part of the definition
	Kohn A et al.^31^	1999	BWT (>5 mm) + PSD (>30 mm)	Not assessed	Yes, >5 mm	Yes, ≥30 mm
CTE						
	Dane B et al.^34^	2023	LN + PSD ≥30 mm	Yes	Evaluated but not part of the definition	Yes, ≥30 mm
	Meng J et al.^36^	2022	LN (≤50%) + BWT (>25%)	Yes, ≤50%	Yes, >25%	Evaluated (>30 mm), but not part of definition
	Duan M et al.^51^	2022	LN (≤50%)	Yes, ≤50%	Evaluated, but not part of definition	Evaluated, but not part of definition
	Hong JT et al.^37^	2022	LN + BWT; PSD optional	Yes	Yes	Evaluated (>30 mm), but not part of definition
	Dane B et al.^32^	2021		Evaluated, but not part of definition	Evaluated, but not part of definition	Not assessed
	Li XH et al.^35^	2021	LN (≤50%) + BWT (>25%)	Yes, ≤50%	Yes, >25%	Not assessed
	Stocker D et al.^38^	2021	LN (≤50%); PSD optional (>30 mm)	Yes, ≤50%	Evaluated, but not part of definition	Evaluated (>30 mm), but not part of definition
	Saade C et al.^39^	2019	LN + PSD	Yes	Evaluated, but not part of definition	Yes
	Pellino G et al.^40^	2016		Not assessed	Yes, >3 mm	Not assessed
	Chiorean MV et al.^24^	2007	LN (≤50%)	Yes, ≤50%	Yes	Yes
	Vogel J et al.^41^	2007	LN + BWT + PSD	Yes, <10 mm	Yes, >5 mm	Evaluated (>30 mm), but not part of definition
MRE						
	Dane B et al.^34^	2023	LN + PSD (≥30 mm)	Yes	Evaluated, but not part of the definition	Yes, ≥30 mm
	Scharitzer M et al.^42^	2023	LN (≤50%) + BWT (≥25%) + PSD (>30 mm)	Yes, ≤50%	Yes, ≥25%	Yes, ≥30 mm
	Servais L et al.^22^	2022		Not assessed	Evaluated, but not part of definition	Evaluated, but not part of definition
	Loch FN et al.^49^	2022	LN (≤50%) + BWT (≥25%) + PSD (>30 mm)	Yes, ≤50%	Yes, ≥25%	Yes, ≥30 mm
	Hong JT et al.^37^	2022	LN + BWT; PSD optional	Yes	Yes	Evaluated (>30 mm), but not part of definition
	Stocker D et al.^38^	2021	LN (≤50%); PSD optional (>30 mm)	Yes, ≤50%	Evaluated, but not part of definition	Evaluated (>30 mm), but not part of definition
	Foti PV et al.^43^	2021	LN	Yes	Evaluated, but not part of definition	Evaluated, but not part of definition
	Fang ZN et al.^44^	2020	LN + PSD	Yes	Evaluated, but not part of definition	Evaluated, but not part of definition
	Barat M et al.^45^	2019	LN (<10 mm); PSD optional (>30 mm)	Yes, <10 mm	Evaluated, but not part of definition	Evaluated, but not part of definition
	Viganò L et al.^23^	2019		Not assessed	Evaluated, but not part of definition	Not assessed
	Pous-Serrano S et al.^46^	2017		Evaluated, but not part of definition	Evaluated, but not part of definition	Evaluated, but not part of definition
	Pellino G et al.^40^	2016		Not assessed	Yes, >3 mm	Not assessed
	Kumar S et al.^25^	2015	BWT	Evaluated, but not part of definition	Yes, >3 mm	Evaluated, but not part of definition
	Spinelli A et al.^34^	2014	LN; PSD optional	Yes	Evaluated, but not part of the definition	Evaluated but not part of the definition
	Sinha R et al.^47^	2013		Not assessed	Yes, >3 mm	Yes
	Ha CY et al.^48^	2011	LN + BWT	Yes	Yes	Not assessed

Lumen narrowing is defined compared to a normally distending proximal segment.

PSD, pre-stenotic dilation; LN, luminal narrowing; BWT, bowel wall thickening.

When limiting the analysis to papers excluding pre-stenotic dilation from the stricture definition, pooled sensitivity and specificity of IUS technique were 0.89 (95% CI, 0.82–0.93) and 0.85 (95% CI, 0.78–0.91), respectively.

Exclusion of studies at higher risk of bias did not substantially alter the findings, with pooled sensitivity and specificity estimates remaining closely aligned with the overall analysis (Supplementary Table S3).

### Definition of stricture and diagnostic accuracy of CTE

**Fig. 3:**
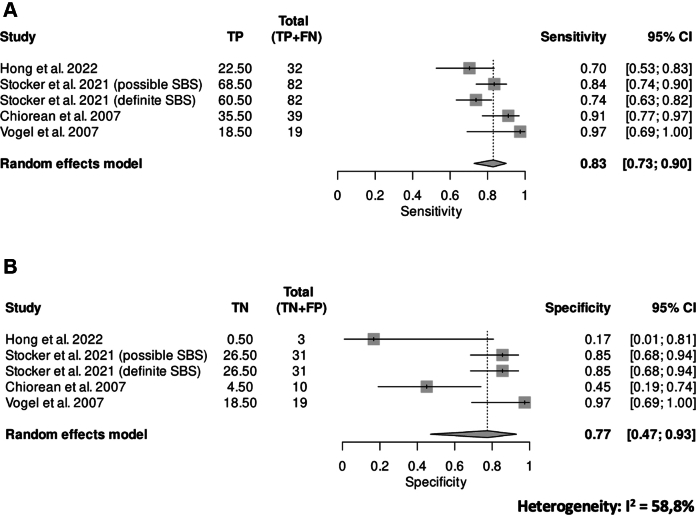
**Pooled diagnostic accuracy of computed tomography entero-graphy**. Pooled diagnostic accuracy of CTE showing sensitivity of 0.83, specificity of 0.77, a modest DOR (11.71), and substantial heterogeneity (*I*^2^ = 58.8%).

9/11 studies provided a definition for stricture^24^^,^^33^^,^^36^, ^37^, ^38^, ^39^^,^^41^^,^^50^^,^^51^ (Table 2). In the included studies, the three primary criteria for defining stricture were luminal narrowing (less than 10 mm, <50% as compared to normal adjacent loops, or unspecified), increased bowel wall thickening (>3 mm, >5 mm, >25%, or unspecified), and/or pre-stenotic dilation (≥30 mm, or unspecified) (Table 2). In detail, luminal narrowing was considered a defining criterion for stricture in 9 out of 11 studies (82%),^24^^,^^33^^,^^36^, ^37^, ^38^, ^39^^,^^41^^,^^50^^,^^51^ with five (45%) specifying a narrowing of ≤50%.^24^^,^^35^^,^^36^^,^^38^^,^^51^ Increased bowel wall thickening was regarded as a defining criterion for stricture in 6 out of 11 studies (54.5%),^24^^,^^35^, ^36^, ^37^^,^^40^^,^^41^ with three different cut-offs (>3 mm, >5 mm or > 25%); in the remaining studies, bowel wall thickening was consistently assessed but not explicitly included as a defining parameter. Pre-stenotic dilation upstream of the stenosis was considered a defining criterion for stricture in only 3 out of 11 studies (27%),^24^^,^^33^^,^^39^ assessed but deemed optional in 5 out of 11 (45%),^36^, ^37^, ^38^^,^^41^^,^^51^ and not evaluated in 3 studies (27%).^32^^,^^40^^,^^50^ Four studies (36%) were suitable for meta-analysis,^24^^,^^37^^,^^38^^,^^41^ the remaining studies were excluded because of insufficient and/or non-comparable data. Pooled sensitivity and specificity for CTE were 0.83 (95% CI, 0.73–0.90) and 0.77 (95% CI, 0.47–0.93), respectively (Fig. 3). A modest DOR was found 11.71 (95% CI, 1.98–69.10), while heterogeneity of studies was high (*I*^2^ = 58.8%).

### Definition of stricture and diagnostic accuracy of MRE

**Fig. 4:**
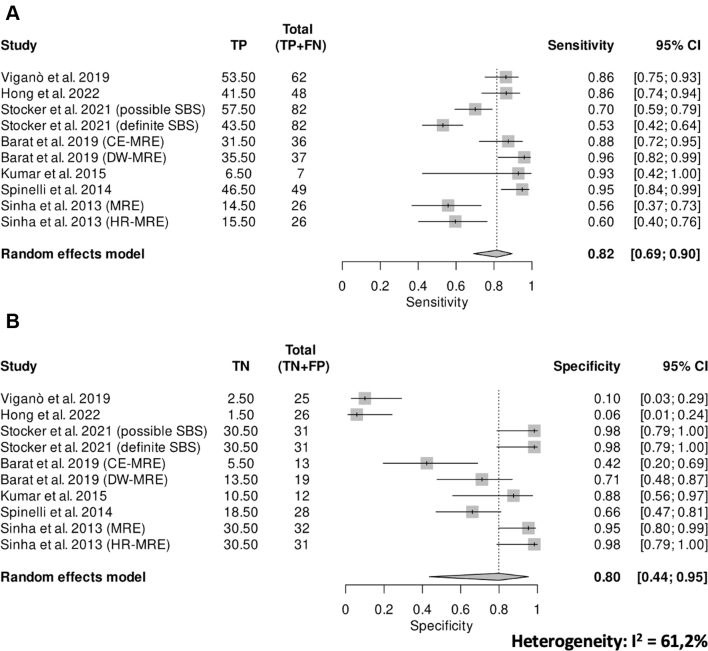
**Pooled diagnostic accuracy of magnetic resonance entero-graphy**. Pooled diagnostic accuracy of MRE showing sensitivity of 0.82, specificity of 0.80, a DOR of 13.82, and substantial heterogeneity (*I*^2^ = 61.2%).

11/16 studies provided a definition for stricture^25^^,^^33^^,^^34^^,^^37^^,^^38^^,^^42^, ^43^, ^44^, ^45^^,^^48^^,^^49^ (Table 2). In the included studies, the three primary criteria for defining stricture were luminal narrowing (less than 10 mm, <50% as compared to normal adjacent loops, or unspecified), increased bowel wall thickening (>3 mm, >25%, or unspecified), and/or pre-stenotic dilation (≥25 mm, ≥30 mm, or unspecified). In detail, luminal narrowing was considered a defining criterion for stricture in 10 out of 16 studies (62.5%),^33^^,^^34^^,^^37^^,^^38^^,^^42^, ^43^, ^44^, ^45^^,^^48^^,^^49^ with three (18%) specifying a narrowing of ≤50%.^38^^,^^42^^,^^49^ Increased bowel wall thickening was regarded as a defining criterion for stricture in 7 out of 16 studies (44%),^25^^,^^37^^,^^40^^,^^42^^,^^47^, ^48^, ^49^ with two different cut-offs (>3 mm, or > 25%); in the remaining studies, bowel wall thickening was consistently assessed but not explicitly included as a defining parameter. Pre-stenotic dilation upstream of the stenosis was considered a defining criterion for stricture in only 4 out of 16 studies (25%),^33^^,^^42^^,^^47^^,^^49^ assessed but deemed optional in 9 out of 16 (56%),^22^^,^^25^^,^^34^^,^^37^^,^^38^^,^^43^, ^44^, ^45^, ^46^ and not evaluated in 3 studies (18%).^23^^,^^40^^,^^48^ Seven studies (44%) were suitable for meta-analysis,^23^^,^^25^^,^^34^^,^^37^^,^^38^^,^^45^^,^^47^ the remaining studies were excluded because of insufficient and/or non-comparable data. Pooled sensitivity and specificity for MRE were 0.82 (95% CI, 0.69–0.90) and 0.80 (95% CI, 0.44–0.95), respectively (Fig. 4), with a DOR equal to 13.82 (95% CI, 4.53–42.14). A significant amount of heterogeneity among studies was present (*I*^2^ = 61.2%).

Exclusion of studies at higher risk of bias yielded a borderline reduction in sensitivity (−5.3%), while specificity remained stable, indicating only a limited influence on the overall conclusions (Supplementary Table S3).

### Comparison of definitions and comparison between imaging modalities

**Fig. 5:**
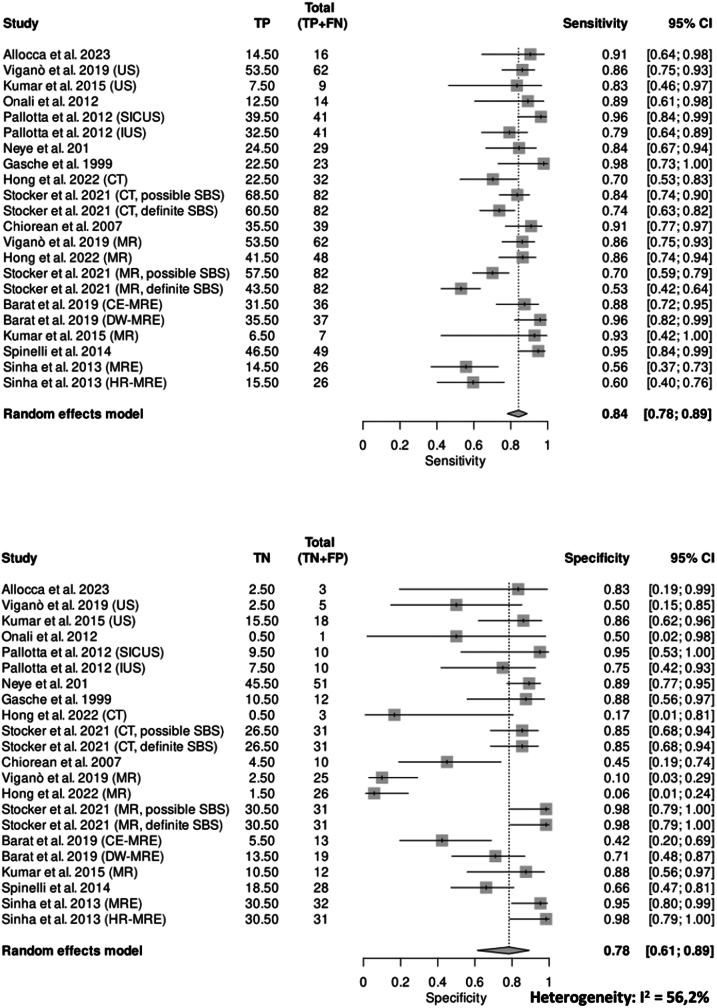
**Pooled diagnostic accuracy of imaging modalities excluding pre-stenotic dilation**. The meta-analysis showed sensitivity of 0.84, specificity of 0.78, a DOR of 15.45, and substantial heterogeneity (*I*^2^ = 56.2%).

The meta-analysis assessing the sensitivity and specificity of all imaging modalities (IUS, MRE, and CTE), including only data where pre-stenotic dilation was excluded from the stricture definition, revealed a pooled sensitivity of 0.84 (95% CI, 0.78–0.89) and a pooled specificity of 0.78 (95% CI, 0.61–0.89), with a diagnostic odds ratio (DOR) of 15.45 (95% CI, 7.58–31.51) (Fig. 5). The I-squared index highlighted a high heterogeneity between studies (*I*^2^ = 56.2%).

A comparative analysis of MRE, SICUS, and IUS in detecting strictures based on number needed to test, assuming a 50% prevalence of stricture in the IBD patient population, showed that SICUS had the highest performances, but differences with both IUS and MRE were not statistically significant.

In fact, the NNT of SICUS was assessed as 2.16, compared to 2.45 of MRE (p = 0.08), as well as the comparison between MRE and IUS showed that both modalities had similar NTTs values (2.45 vs. 2.3) (p = 0.51). Finally, the comparison between SICUS and IUS indicated that SICUS did not statistically overperform IUS (2.16 vs. 2.33) (p = 0.29).

Leave-one-out sensitivity analyses revealed variations in the pooled estimates upon sequential exclusion of individual studies; however, these did not alter the overall direction or interpretation of the results. Specificity varied by more than 5% following the exclusion of two studies (10.2% and 7.2%, respectively). Detailed sensitivity and specificity estimates for each iteration are provided in Supplementary Table S4.

Using GRADE, certainty of the evidence for detection of small-bowel strictures was rated moderate for US and MRE, and low for CTE (Supplementary Table S5).

## Discussion

This systematic review and meta-analysis assessed the definition criteria and diagnostic performance characteristic of cross-sectional imaging modalities for detecting CD-associated strictures. Despite substantial heterogeneity in the diagnostic criteria for strictures, cross-sectional imaging demonstrated consistently high accuracy, with sensitivity and specificity exceeding 80% across all modalities. Our primary objective was to ensure the highest methodological rigor by exclusively using histology as the reference standard and updating the subgroup analysis originally conducted by Bettenworth et al.^14^ incorporating histological confirmation as the diagnostic benchmark.

Our systematic review underscores the considerable variability in the criteria used to diagnose small bowel structures in CD (Table 2). Different studies have employed varying combinations of bowel wall thickness, luminal narrowing, and pre-stenotic dilation to define strictures, with most incorporating only one or two of these parameters. This variability may reflect differences in clinical practice, study design, imaging equipment, and patient populations. In particular, the inconsistent reporting of pre-stenotic dilation—especially in ultrasonography-based assessments—could be partially attributed to the absence of oral contrast agents and fasting protocols typically used in IUS, which may limit small bowel distension and reduce the visibility of upstream dilation.

Key findings of our study were the inclusion of the sub-analysis for SICUS, and the sub-analysis excluding pre-stenotic dilation. Notably, IUS, MRE, and CTE maintained high sensitivity for detecting strictures even in the absence of pre-stenotic dilation [pooled sensitivity for IUS/MRE/CTE of 0.84 (95% CI, 0.78–0.89) and pooled specificity of 0.75 (95% CI, 0.58–0.87)], indicating that the imaging parameters of bowel wall thickening and luminal narrowing may be sufficient for accurate diagnosis. Pre-stenotic dilation may not be an essential criterion for the radiological or ultrasound-based diagnosis of strictures, and its diagnostic thresholds (≥25 mm, ≥30 mm, >50% relative to an adjacent intestinal loop) have yet to be validated through future clinical research, especially for IUS. Thus, given that pre-stenotic dilation does not appear essential for the diagnosis of strictures, this finding could simplify diagnostic protocols and reduce reliance on more complex imaging techniques.

Among the modalities compared, SICUS demonstrated a potential advantage over MRE and IUS in stricture detection, though this difference was not statistically significant. The lack of a statistically significant difference in diagnostic accuracy for SICUS, combined with its limited cost-effectiveness, reduces its overall clinical appeal as a preferred imaging modality. Similarly, MRE and IUS exhibited comparable diagnostic performance, with a slight, non-significant advantage for IUS, suggesting that the choice between these modalities should be guided by availability and clinical considerations. Consistent with our analysis, CTE appears to demonstrate slightly lower diagnostic accuracy. Still, given the limited number of studies included in the evaluation of this modality, we can conclude for substantial overlap of CTE with the other methods.

In summary, the choice between IUS, MRE or CTE for the diagnosis and assessment of CD-associated stricture should be based on factors such as cost, availability, and clinical context, rather than on their diagnostic accuracy alone. Emerging modalities—including ultrasound elastography, magnetisation transfer (MT) imaging, and diffusion-weighted imaging (DWI) MRI—have demonstrated promising results in preliminary studies for distinguishing fibrotic from inflammatory strictures,^44^^,^^52^^,^^53^ with MT and DWI showing moderate to strong correlation with histopathological fibrosis scores.^53^^,^^54^ Despite their potential to refine stricture characterization and improve clinical decision-making, these advanced techniques remain predominantly research tools and are not yet standardised or broadly integrated into routine clinical algorithms.

**Fig. 6:**
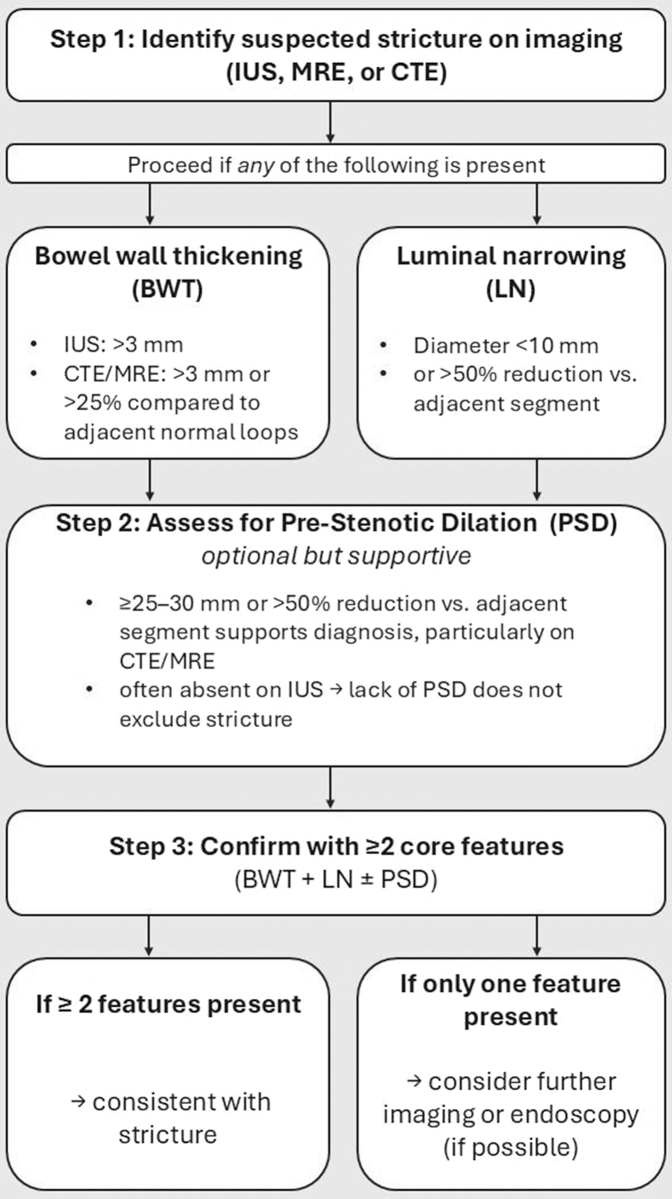
**Proposed imaging-based algorithm for stricture diagnosis**. A stepwise approach combining bowel wall thickening, luminal narrowing, and (optionally) pre-stenotic dilation is suggested to guide the non-invasive diagnosis of small bowel strictures.

Our results are in line with those found in the literature,^11^^,^^12^^,^^14^^,^^15^ however, our analysis extended to all cross-sectional methods and was not limited to one method.^12^ A further strength of our work was that, in addition to the qualitative and synthetic analysis,^11^^,^^14^ we performed meta-analyses assessing pooled sensitivity and specificity of the included works. A relevant finding of our meta-analysis is that in case of the absence of pre-stenotic dilation, which can often occur in clinical practice (i.e., asymptomatic strictures, chronic strictures, fasting and lack of oral contrast in IUS), strictures can still reliably be diagnosed, with both sensitivity and specificity ≥80% for all techniques. Based on our findings, we propose a pragmatic imaging-based algorithm to guide the non-invasive diagnosis of small bowel strictures (Fig. 6). We acknowledge some possible limitations of our study, despite the rigor in using histology as the reference standard, some studies included in the meta-analysis may have employed varying histological criteria, potentially introducing bias. Additionally, the limited number of studies for certain imaging modalities, such as CTE, may affect the generalizability of our findings. We acknowledge that including only studies with surgical histopathology as the reference standard may introduce selection bias, as patients undergoing surgery often represent more severe disease; however, this approach was deliberately chosen to ensure high diagnostic accuracy and a consistent reference standard across imaging modalities. Deliberately, our study focused on small bowel strictures, as colonic strictures are inconsistently detected by cross-sectional imaging—particularly without colonic contrast—and their definitions are even less standardised. This approach allowed for a more focused and homogeneous analysis, although it represents a limitation in not addressing strictures in other intestinal segments. To maintain methodological transparency, we conducted a scoping search of PubMed and Embase for the period January 1, 2025, to July 15, 2025, using the same search strategy. No large-scale trials or methodologically novel studies were identified that would be expected to significantly impact our findings or alter the strength of the conclusions presented.

Leave-one-out sensitivity analyses indicated that no single study unduly influenced the overall results. Specificity shifted by more than 5% with the exclusion of two studies (10.2% and 7.2%, respectively), but these changes did not affect the qualitative interpretation of the pooled estimates. Subgroup analyses stratified by imaging modality showed that exclusion of higher-risk studies had minimal impact on ultrasound results, whereas in the MRE subgroup sensitivity was modestly reduced (−5.3 percentage points) with stable specificity. Such analysis was not conducted for CTE, as none of the included studies was at high risk of bias. Overall, these findings support the robustness of the main results, while highlighting the greater susceptibility of MRE estimates to variability due to the smaller evidence base.

Finally, although stricture length may hold pathophysiological and clinical relevance, it was inconsistently defined or not reported across the included studies. Notably, current consensus guidelines for the imaging-based diagnosis of CD-associated strictures do not include stricture length as a required criterion; however, its potential role remains insufficiently explored and may warrant further investigation in future research.

In conclusion, this work lays the groundwork for the development of future indices, which remains a crucial obstacle to the advancement of standardization of definition and assessment of CD-associated strictures. Future research should focus on validating diagnostic thresholds, particularly for pre-stenotic dilation, and exploring the clinical relevance of combining imaging modalities.

## Contributors

MA conceived the article. ADB, SB, GM, AB and wrote the article. ADB, FF, SB, GM and AB created tables and figures. FF performed the statistical analysis. MA supervised the project. ADB, FF and AB assessed the methodology of the paper. ADB, FF, and MA had access to and verified the underlying study data. SD, DB, AA and MA critically reviewed the content of the paper and edited the final draft. The manuscript was approved by all authors.

Guarantor of the article: Mariangela Allocca.

## Data sharing statement

The data underlying this article are available in its online Supplementary Material and upon request to the corresponding author and will be made available with publication.

## Declaration of interests

**SD** has served as a speaker, consultant and advisory board member for Schering- Plough, AbbVie, MSD, UCB Pharma, Ferring, Cellerix, Millennium Takeda, Nycomed, Pharmacosmos, Actelion, Alphawasserman, Genentech, Grunenthal, Pfizer, Astra Zeneca, Novo Nordisk, Cosmo Pharmaceuticals, Vifor and Johnson & Johnson, Nikkiso Europe GMBH, Theravance. **AA** has received consulting fees from AbbVie, Allergan, Amgen, Arena, Biogen, Boehringer Ingelheim, Bristol-Myers Squibb, Celgene, Celltrion, Eli-Lilly, Ferring, Galapagos, Gilead, Janssen, MSD, Mylan, Pfizer, Protagonist Therapeutics, Roche, Samsung Bioepis, Sandoz and Takeda; speaker's fees from AbbVie, Amgen, Arena, Biogen, Bristol-Myers Squibb, Eli-Lilly, Ferring, Galapagos, Gilead, Janssen, MSD, Novartis, Pfizer, Roche, Samsung Bioepis, Sandoz, Takeda, and Tigenix; and research support from Biogen, MSD, Takeda, and Pfizer. **ADB** has received speaker's fees from AbbVie, Galapagos, Ferring and Celltrion. **MA** received consulting fees from Nikkiso Europe, Mundipharma, Janssen, Abbvie, Ferring, Galapagos, Lilly, Alfasigma, Sandoz and Pfizer. **FF**, **SB**, **GM**, and **AB** declare no competing interests.

**DB** served as advisory board member or consultant for AbbVie, Alfasigma, Amgen, Arena, Art tempi, BNG Service GmbH, Bristol Myers Squibb, CED-Service GmbH, Celltrion, Doctorflix, DGVS, Diaplan, Else Kröner-Fresenius Foundation, Falk Foundation, Fresenius, Galapagos, Gastro Today, GSK, Guidepoint, Hexal, Impulze, Ferring, Janssen Cilag, Lilly, Medical Tribune, MedTriX, MSD, Mylan, Onkowissen, Pharmacosmos, Pfizer, Roche, Sandoz, Stada, StreamedUp, Takeda, Tetrameros, Thieme, Tillotts Pharma, UCB Biopiharma, Viatris and Vifor Pharma.

## Appendix A Supplementary data

Supplementaries Combined

Caption for Supplementary Material

## References

[1] Dolinger M., Torres J., Vermeire S. (2024). Crohn's disease. Lancet.

[2] Thia K.T., Sandborn W.J., Harmsen W.S., Zinsmeister A.R., Loftus E.V. (2010). Risk factors associated with progression to intestinal complications of Crohn's disease in a population-based cohort. Gastroenterology.

[3] Rieder F., Mukherjee P.K., Massey W.J., Wang Y., Fiocchi C. (2024). Fibrosis in IBD: from pathogenesis to therapeutic targets. Gut.

[4] Lunder A.K., Bakstad L.T., Jahnsen J. (2019). Assessment of bowel inflammation and strictures by magnetic resonance enterography in long-term Crohn's disease. J Crohns Colitis.

[5] Bouhnik Y., Carbonnel F., Laharie D. (2018). Efficacy of adalimumab in patients with Crohn's disease and symptomatic small bowel stricture: a multicentre, prospective, observational cohort (CREOLE) study. Gut.

[6] Allocca M., Bonifacio C., Fiorino G. (2017). Efficacy of tumour necrosis factor antagonists in stricturing Crohn's disease: a tertiary center real-life experience. Dig Liver Dis.

[7] Schulberg J.D., Wright E.K., Holt B.A. (2022). Intensive drug therapy versus standard drug therapy for symptomatic intestinal Crohn's disease strictures (STRIDENT): an open-label, single-centre, randomised controlled trial. Lancet Gastroenterol Hepatol.

[8] Frolkis A.D., Dykeman J., Negrón M.E. (2013). Risk of surgery for inflammatory bowel diseases has decreased over time: a systematic review and meta-analysis of population-based studies. Gastroenterology.

[9] Maaser C., Sturm A., Vavricka S.R. (2019). ECCO-ESGAR guideline for diagnostic assessment in IBD Part 1: initial diagnosis, monitoring of known IBD, detection of complications. J Crohns Colitis.

[10] Allocca M., Furfaro F., Fiorino G., Peyrin-Biroulet L., Danese S. (2021). Point-of-care ultrasound in inflammatory bowel disease. J Crohns Colitis.

[11] Lu C., Rosentreter R., Delisle M. (2024). Systematic review: defining, diagnosing and monitoring small bowel strictures in Crohn's disease on intestinal ultrasound. Aliment Pharmacol Ther.

[12] Pruijt M.J., De Voogd F.A.E., Montazeri N.S.M., Van Etten-Jamaludin F.S., D'Haens G.R., Gecse K.B. (2024). Diagnostic accuracy of intestinal ultrasound in the detection of intra-abdominal complications in Crohn's disease: a systematic review and meta-analysis. J Crohns Colitis.

[13] Bettenworth D., Baker M.E., Fletcher J.G. (2024). A global consensus on the definitions, diagnosis and management of fibrostenosing small bowel Crohn's disease in clinical practice. Nat Rev Gastroenterol Hepatol.

[14] Bettenworth D., Bokemeyer A., Baker M. (2019). Assessment of Crohn's disease-associated small bowel strictures and fibrosis on cross-sectional imaging: a systematic review. Gut.

[15] Rieder F., Bettenworth D., Ma C. (2018). An expert consensus to standardise definitions, diagnosis and treatment targets for anti-fibrotic stricture therapies in Crohn's disease. Aliment Pharmacol Ther.

[16] Guglielmo F.F., Anupindi S.A., Fletcher J.G. (2020). Small bowel Crohn disease at CT and MR enterography: imaging atlas and glossary of terms. Radiographics.

[17] Cumpston M., Li T., Page M.J. (2019). Updated guidance for trusted systematic reviews: a new edition of the Cochrane handbook for systematic reviews of interventions. Cochrane Database Syst Rev.

[18] Hutton B., Salanti G., Caldwell D.M. (2015). The PRISMA extension statement for reporting of systematic reviews incorporating network meta-analyses of health care interventions: checklist and explanations. Ann Intern Med.

[19] Page M.J., McKenzie J.E., Bossuyt P.M. (2021). The PRISMA 2020 statement: an updated guideline for reporting systematic reviews. BMJ.

[20] Whiting P.F., Rutjes A.W.S., Westwood M.E. (2011). QUADAS-2: a revised tool for the quality assessment of diagnostic accuracy studies. Ann Intern Med.

[21] Allocca M., Dal Buono A., D'Alessio S. (2023). Relationships between intestinal ultrasound parameters and histopathologic findings in a prospective cohort of patients with Crohn's disease undergoing surgery. J Ultrasound Med.

[22] Servais L., Boschetti G., Meunier C. (2022). Intestinal conventional ultrasonography, contrast-enhanced ultrasonography and magnetic resonance enterography in assessment of Crohn's disease activity: a comparison with surgical histopathology analysis. Dig Dis Sci.

[23] Viganò L., Mineccia M., Bertolino F. (2019). Intraoperative ultrasonography in patients undergoing surgery for Crohn's disease. Prospective evaluation of an innovative approach to optimize staging and treatment planning. Updates Surg.

[24] Chiorean M.V., Sandrasegaran K., Saxena R., Maglinte D.D., Nakeeb A., Johnson C.S. (2007). Correlation of CT enteroclysis with surgical pathology in Crohn's disease. Am J Gastroenterol.

[25] Kumar S., Hakim A., Alexakis C. (2015). Small intestinal contrast ultrasonography for the detection of small bowel complications in Crohn's disease: correlation with intraoperative findings and magnetic resonance enterography. J Gastroenterol Hepatol.

[26] Onali S., Calabrese E., Petruzziello C. (2012). Small intestine contrast ultrasonography vs computed tomography enteroclysis for assessing ileal Crohn's disease. World J Gastroenterol.

[27] Pallotta N., Vincoli G., Montesani C. (2012). Small intestine contrast ultrasonography (SICUS) for the detection of small bowel complications in Crohn's disease: a prospective comparative study versus intraoperative findings. Inflamm Bowel Dis.

[28] Neye H., Ensberg D., Rauh P. (2010). Impact of high-resolution transabdominal ultrasound in the diagnosis of complications of Crohn's disease. Scand J Gastroenterol.

[29] Parente F., Maconi G., Bollani S. Bowel ultrasound in assessment of Crohn's disease and detection of related small bowel strictures: a prospective comparative study versus x ray and intraoperative findings. http://www.gutjnl.com.

[30] Gasche C., Moser G., Turetschek K., Schober E., Moeschl P., Oberhuber G. (1999).

[31] Kohn A., Cerro T., Milite G., De Angelis E., Prantera C. (1999). prospective evaluation of transabdominal bowel sonography in the diagnosis of intestinal obstruction in Crohn’ s disease: comparison with plain abdominal film and small bowel enteroclysis. Inflamm Bowel Dis.

[32] Dane B., Sarkar S., Nazarian M. (2021). Crohn disease active inflammation assessment with iodine density from dual-energy CT enterography: comparison with histopathologic analysis. Radiology.

[33] Dane B., Remzi F.H., Grieco M. (2023). Preoperative cross-sectional imaging findings in patients with surgically complex ileocolic Crohn's disease. Abdom Radiol (NY).

[34] Spinelli A., Fiorino G., Bazzi P. (2014). Preoperative magnetic resonance enterography in predicting findings and optimizing surgical approach in Crohn's disease. J Gastrointest Surg.

[35] Li X., Liang D., Meng J. (2021). Development and validation of a novel computed-tomography enterography radiomic approach for characterization of intestinal fibrosis in Crohn's disease. Gastroenterology.

[36] Meng J., Mao Y., Zhou J. (2022). Mesenteric abnormalities play an important role in grading intestinal fibrosis in patients with Crohn's disease: a computed tomography and clinical marker-based nomogram. Therap Adv Gastroenterol.

[37] Hong J.T., Kutaiba N., Parameswaran B. (2022). Sensitivity of pre-operative imaging and radiologist inter-rater reliability in detecting lesions in Crohn's disease. ANZ J Surg.

[38] Stocker D., King M.J., El Homsi M. (2021). Luminal narrowing alone allows an accurate diagnosis of Crohn's disease small bowel strictures at cross-sectional imaging. J Crohns Colitis.

[39] Saade C., Nasr L., Sharara A. (2019). Crohn's disease: a retrospective analysis between computed tomography enterography, colonoscopy, and histopathology. Radiography.

[40] Pellino G., Nicolai E., Catalano O.A. (2016). PET/MR versus PET/CT imaging: impact on the clinical management of small-bowel Crohn's disease. J Crohns Colitis.

[41] Vogel J., Da Luz Moreira A., Baker M. (2007). CT enterography for Crohn's disease: accurate preoperative diagnostic imaging. Dis Colon Rectum.

[42] Scharitzer M., Macher-Beer A., Mang T. (2023). Evaluation of intestinal fibrosis with 68Ga-FAPI PET/MR enterography in Crohn disease. Radiology.

[43] Foti P.V., Travali M., Farina R. (2021). Can conventional and diffusion-weighted mr enterography biomarkers differentiate inflammatory from fibrotic strictures in Crohn's disease?. Medicina (Lithuania).

[44] Fang Z.N., Li X.H., Lin J.J. (2020). Magnetisation transfer imaging adds information to conventional MRIs to differentiate inflammatory from fibrotic components of small intestinal strictures in Crohn's disease. Eur Radiol.

[45] Barat M., Hoeffel C., Bouquot M. (2019). Preoperative evaluation of small bowel complications in Crohn's disease: comparison of diffusion-weighted and contrast-enhanced MR imaging. Eur Radiol.

[46] Pous-Serrano S., Frasson M., Palasí Giménez R. (2017). Accuracy of magnetic resonance enterography in the preoperative assessment of patients with Crohn's disease of the small bowel. Colorectal Dis.

[47] Sinha R., Murphy P., Sanders S. (2013). Diagnostic accuracy of high-resolution MR enterography in Crohn's disease: comparison with surgical and pathological specimen. Clin Radiol.

[48] Ha C.Y., Kumar N., Raptis C.A., Narra V.R., Ciorba M.A. (2011). Magnetic resonance enterography: safe and effective imaging for stricturing Crohn's Disease. Dig Dis Sci.

[49] Loch F.N., Kamphues C., Beyer K. (2022). Diagnostic accuracy of magnetic resonance enterography for the evaluation of active and fibrotic inflammation in Crohn's disease. Front Surg.

[50] Li X.H., Feng S.T., Cao Q.H. (2021). Degree of creeping fat assessed by computed tomography enterography is associated with intestinal fibrotic stricture in patients with Crohn's disease: a potentially novel mesenteric creeping fat index. J Crohns Colitis.

[51] Duan M., Guan B., Cao L. (2022). Computed tomography enterography predicts surgical-free survival in symptomatic stricturing Crohn's disease. Abdom Radiol (NY).

[52] Dal Buono A., Faita F., Peyrin-Biroulet L., Danese S., Allocca M. (2022). Ultrasound elastography in inflammatory bowel diseases: a systematic review of accuracy compared with histopathological assessment. J Crohns Colitis.

[53] Li Z., Chen Z., Zhang R. (2024). Comparative analysis of [18F]F-FAPI PET/CT, [18F]F-FDG PET/CT and magnetization transfer MR imaging to detect intestinal fibrosis in Crohn's disease: a prospective animal model and human cohort study. Eur J Nucl Med Mol Imaging.

[54] Kim P.H., Yoon H.M., Jung A.Y., Lee J.S., Cho Y.A. (2022). Diagnostic performance of diffusion-weighted imaging for evaluation of bowel inflammation in paediatric inflammatory bowel disease: a systematic review and meta-analysis. J Crohns Colitis.

