# Supplementary material for: Variability and performance of radiologic stricture parameters in Crohn's disease: a systematic review and meta-analysis
Source: eClinicalMedicine. 2025 Oct 8;89:103541. doi: 10.1016/j.eclinm.2025.103541 (PMC12538911; doi:10.1016/j.eclinm.2025.103541)
Supplement: Supplementaries Combined [file mmc1.pdf]

Supplementary materials:

The detailed primary search strategy for the systematic search is displayed in **Supplementary Table 1**. was implemented by using combinations of the specific items available in the database used: medical subject headings (MeSH) terms and Title and Abstracts (tiab) and keywords for pubmed/MEDLINE; Exploded (*/Exp*) and title and abstract syntax (:ti,ab,kw) for Embase. Boolean operators OR, NOT, AND and NEXT were used to create the search string. The present systematic review was performed with the aim of updating the previous systematic analysis of Bettenworth et al.(Bettenworth et al. 2019). Concerning the evaluation of strictures with ultrasonography, a recent systematic review was published, and the results of our systematic search were cross-checked to improve the accuracy of our analysis(Lu et al. 2024). The search on the 3 databases was nevertheless performed from inception, re-checking all full papers at inclusion, updated until January 2024. Hand-searching of additional papers was performed by manual consultation of available systematic reviews(Lu et al. 2024; Rieder et al. 2018; Bettenworth et al. 2019) on the topic within the inclusion criteria and the overall outcome of the paper. To perform the systematic review, every title and abstract was uploaded into the web tool Rayyan (*Rayyan – Intelligent Systematic Review - Rayyan*)(Ouzzani et al. 2016). The research for duplicates was handmade in order not to incur automated tool biases. The full paper's evaluation was performed by 3 independent authors (SB, GM and AB) after retrieving every article on Microsoft Excel Spreadsheet (*XP Professional edition; Microsoft, Redmond, Washington, USA*). Every reviewer independently blindly assessed all full texts applying the inclusion and exclusion criteria, grading every paper "yes" (for inclusion), "no" (for exclusion with exclusion reasons) and "maybe" in case of uncertainty. After unblinding, the reviewers discussed the uncertainties or the discrepancies and found agreement with inclusion or exclusion of the paper. In case of further discordance, a fourth reviewer (ADB) was consulted. Inclusion and exclusion criteria for the systematic search were decided with the purpose of excluding all papers not reporting a clear reference standard for CD-related diagnosis. Furthermore, with the aim of identifying potential heterogeneity in the

radiological assessment of CD strictures, particularly for the cut-off previously identified and provided by a panel of experts (Rieder et al. 2018)(Panes et al. 2013) for pre-stenotic dilation (PSD), luminal narrowing (LN) and Bowel Wall Thickness (BWT). The detailed major exclusion decisions are displayed in **Figure 1.**

Supplementary Table 1 Detailed search strategy for systematic review

Search	Query	Results
**Pubmed/MEDLINE**		
#4	Search: **#1 AND #2 AND #3**	3,203
#3	NOT ("Review"[Publication Type] OR "Systematic Review" [Publication Type] OR "Meta-Analysis"[Publication Type] OR "Meta-Analysis as Topic"[Mesh] OR “meta-analysis”[tiab] OR “systematic review*”[tiab] OR “systematic literature review*”[tiab] OR "Letter"[Publication Type] OR "Editorial"[Publication Type] OR "Comment"[Publication Type] OR "Case Reports"[Publication Type] OR "Interview" [Publication Type])	7,748,984
#2	("Magnetic Resonance Imaging"[Mesh] OR "Tomography, Emission-Computed"[Mesh] OR "Ultrasonography"[Mesh] OR “Cross-sectional”[tiab] OR “Cross-sectional imag*”[tiab] OR “enterography”[tiab] OR “MRE”[tiab] OR “CTE”[tiab] OR “MRI”[tiab] OR “PET-CT”[tiab] OR “MR-enteroclysis”[tiab] OR “enteroclysis”[tiab] OR “intestinal ultrasound”[tiab] OR “bowel ultrasound”[tiab] OR “elastography”[tiab] OR “ultrasonography”[tiab] OR “CEUS”[tiab] OR “contrast* ultrasonography*”)	1,805,564
#1	"Crohn Disease"[Mesh] OR “Crohn’s disease”[tiab] OR “Crohn”[tiab] OR “Crohn’s Enteritis”[tiab]	67,449
**Embase**		
**Search**	**Query**	**Results**
#4		5,894
#3	('chapter'/it OR 'conference abstract'/it OR 'conference paper'/it OR 'conference review'/it OR 'editorial'/it OR 'erratum'/it OR 'letter'/it OR 'note'/it OR 'preprint'/it OR 'review'/it OR 'short survey'/it OR 'tombstone'/it)	13,228,127

Search	Query	Results
#2	‘Magnetic Resonance Imaging’/exp OR ‘Tomography, Emission-Computed’/exp OR ‘Ultrasonography’/exp OR (“Cross-sectional” OR “Cross-sectional imag*” OR “enterography” OR “MRE” OR “CTE” OR “MRI” OR “PET-CT” OR “MR-enteroclysis” OR “enteroclysis” OR “intestinal ultrasound” OR “bowel ultrasound” OR “elastography” OR “ultrasonography” OR “CEUS” OR “contrast* ultrasonography*”):ti,ab,kw	3,246,229
#1	‘Crohn disease’/exp OR (Crohn* disease OR Crohn OR Crohn* Enteritis):ti,ab,kw	120,794

Cochrane

Search	Query	Results
#3	#1 AND #2	323
#2	(Cross-sectional OR Cross-sectional NEXT imag* OR enterography OR MRE OR CTE OR MRI OR PET-CT OR MR-enteroclysis OR enteroclysis OR intestinal NEXT ultrasound OR bowel NEXT ultrasound OR elastography OR ultrasonography OR CEUS OR contrast* NEXT ultrasonography*):ti,ab,kw	-
#1	(Crohn* NEXT disease OR Crohn OR Crohn* NEXT Enteritis):ti,ab,kw	-

Supplementary Table 2. QUADAS-2 analysis of included studies.

Study	Patient selection			Index test(s)		Reference standard		Flow and timing				Risk of bias
	1	2	3	4	5	6	7	8	9	10	11	
Allocca et al, 2023	☺	☺	☺	☺	☺	☺	☺	☺	☺	☺	☺	Low
Servais et al, 2022	☺	☺	☺	☺	☹	☺	☺	☺	☺	☺	☺	Low
Viganò et al, 2019	☺	☺	☺	☺	☹	☺	☺	?	☺	☺	☺	High
Kumar et al, 2015	☺	☺	☺	☺	☹	☺	☹	☺	☺	☺	☺	High
Onali et al, 2012	☺	☺	☺	☺	☺	☺	☺	☺	☺	☺	☺	Low
Pallotta et al, 2012	☺	☺	☺	☺	☺	☺	?	☺	☺	☺	☺	Unclear
Neye H et al, 2010	☺	☺	☺	☺	☺	☺	☹	☺	☺	☺	☺	Low

Parente F et al, 2002	☺	☺	☺	☺	☺	☺	☺	☺	☺	☺	☺	Low
Gasche C et al, 1999	☺	☺	☺	☺	☹	☺	☺	?	☺	☺	☺	High
Kohn A et al, 1999	☺	☺	☺	☺	☹	☺	☺	☺	☺	☺	☺	Low
Dane B et al, 2023	☺	☺	☺	☺	☹	☺	☺	☺	☺	☺	☺	Low
Meng J et al, 2022	☺	☺	☺	☺	☺	☺	☺	☺	☺	☺	☺	Low
Duan M et al, 2022	☺	☺	☺	☺	☺	☺	☺	☺	☺	☺	☺	Low
Hong JT et al, 2022	☺	☺	☺	☺	☺	☺	?	☺	☺	☺	☹	Low
Dane B et al, 2021	☺	☺	☺	☺	☹	☺	☺	☺	☺	☺	☹	High
Li XH et al, 2021	☺	☺	☺	☺	☺	☺	☺	☺	☺	☺	☺	Low
Stocker D et al, 2021	☺	☺	☺	☺	☺	☺	?	☺	☺	☺	☹	Low
Saade C et al, 2019	☺	☺	☺	☺	☺	☺	☺	☺	☺	☺	☹	Unclear

Pellino G et al, 2016	☺	☺	☺	☺	☹	☺	☺	☺	☺	☺	☹	Low
Chiorean MV et al, 2007	☺	☺	☺	☺	☺	☺	☺	☺	☺	☺	☹	Low
Vogel J et al, 2007	☺	☺	☺	☺	☺	☺	?	☺	☺	☺	☺	Low
Scharitzer M et al, 2023	☺	☺	☺	☺	☺	☺	☺	☺	☺	☺	☺	Low
Loch FN et al, 2022	☺	☺	☺	☺	☺	☺	☺	☺	☺	☺	☺	Low
Foti PV et al, 2021	☺	☺	☺	☺	☹	☺	☺	☺	☺	☺	☺	High
Fang ZN et al, 2020	☺	☺	☺	☺	☹	☺	☺	☺	☺	☺	☺	High
Barat M et al, 2019	☺	☺	☺	☹	☺	☺	☹	☺	☺	☺	☺	Low
Pous- Serrano S et al, 2017	☺	☺	☺	☺	☺	☺	?	☺	☺	☺	☺	Low
Spinelli A et al, 2014	☺	☺	☺	☺	☹	☺	☹	☺	☺	☺	☺	High

Sinha R et al, 2013	☺	☺	☺	☺	☺	☺	☺	☺	☺	☺	☺	Low
Ha CY et al, 2011	☺	☺	☺	☺	☹	☺	☹	☺	☺	☺	☺	High

Abbreviations: ☺=Yes; ☹= No; ?= Unclear;

QUADAS-2 is structured so that 4 key domains are each rated in terms of the risk of bias and the concern regarding applicability to the research question (as defined above). Each key domain has a set of signaling questions to help reach the judgments regarding bias and applicability. 1. Was a consecutive or random sample of patients enrolled? 2. Was a case-control design avoided? 3. Did the study avoid inappropriate exclusions? 4. Were the index test results interpreted without knowledge of the results of the reference standard? 5. If a threshold was used, was it pre-specified? 6. Is the reference standard likely to correctly classify the target condition? 7. Were the reference standard results interpreted without knowledge of the results of the index test? 8. Was there an appropriate interval between index test(s) and reference standard? 9. Did all patients receive a reference standard? 10. Did patients receive the same reference standard? 11. Were all patients included in the analysis?

Supplementary Table 3. Sensitivity analysis excluding studies at high risk of bias

	**All studies**			**Excluding studies with high risk of bias**			**Absolute difference**		
	Sensitivity	Specificity	I2	Sensitivity	Specificity	I2	Sensitivity	Specificity	I2
MRE	82	80	61,2	76,7	84,6	59,2	5,3	-4,6	2
US	88	86	0	86,4	87,2	0	1,6	-1,2	0

Values are pooled sensitivity and specificity (%) with I^2 heterogeneity index. Excluding studies judged at high risk of bias by QUADAS-2 produced minimal absolute differences, indicating robustness of the primary findings.

Supplementary Table 4. Leave-one-out sensitivity analysis.

Excluded paper	sensitivity	specificity	delta sensitivity	delta specificity
Allocca et al. 2023	83,5	75,4	0,5	2,6
Viganò et al. 2019	83,4	79,8	0,6	-1,8
Kumar et al. 2015	83,6	73,4	0,4	4,6
Onali et al. 2012	83,5	76,4	0,5	1,6
Pallotta et al. 2012	82,5	73,3	1,5	4,7
Neye et al. 201	83,7	73,9	0,3	4,1
Gasche et al. 1999	82,9	74,6	1,1	3,4
Hong et al. 2022	84	80,3	0	-2,3
Stocker et al. 2021	86,6	67,8	-2,6	10,2
Chiorean et al. 2007	83,1	76,6	0,9	1,4
Barat et al. 2019	82,1	76,4	1,9	1,6
Spinelli et al. 2014	82,8	75,6	1,2	2,4
Sinha et al. 2013	85,4	70,8	-1,4	7,2

Each study was sequentially omitted from the meta-analysis to assess its influence on pooled estimates. Values are pooled sensitivity and specificity (%) after exclusion, with delta values showing the absolute change from the overall model.

Supplementary Table 5. Summary of Findings (GRADE) for diagnostic accuracy of US, MRE, and CTE in detecting small-bowel Crohn's disease strictures.

Index test (modality)	Population & setting	Reference standard	Outcome	Pooled sensitivity (95% CI)	Pooled specificity (95% CI)	No. of studies	Certainty of evidence (GRADE)	Reasons for rating
Ultrasound (IUS ± SICUS)	Adults with known CD	Surgical histopathology	Detection of small-bowel strictures	0.88 (0.83–0.91)	0.86 (0.79–0.91)	9	Moderate	Downgraded one level for indirectness. No downgrade for inconsistency (I^2=0%).
Magnetic Resonance Enterography (MRE)	Adults with known CD	Surgical histopathology	Detection of small-bowel strictures	0.82 (0.69–0.90)	0.80 (0.44–0.95)	7	Moderate	Downgraded one level for inconsistency (substantial heterogeneity, I^2≈61%). We did not formally downgrade for imprecision, but we highlight the wide specificity CI in the narrative.
Computed Tomography Enterography (CTE)	Adults with known CD	Surgical histopathology	Detection of small-bowel strictures	0.83 (0.73–0.90)	0.77 (0.47–0.93)	4	Low	Downgraded two levels for inconsistency (I^2≈56%) and imprecision (wide CI; small evidence base).

GRADE domains considered: risk of bias (QUADAS-2), inconsistency (I^2, overlap of CIs), indirectness (surgical cohorts; applicability to broader CD populations), imprecision (width of 95% CIs; total information size), publication bias (not formally assessed given small numbers).

Pooled estimates come from the bivariate random-effects model in the primary analysis; IUS includes SICUS where applicable.

Ratings reflect certainty within each modality vs histopathology; they do not rank modalities against each other.

Where heterogeneity and/or CIs were wide, this uncertainty is reflected in the GRADE rating and narrative.
