# Supplementary material for: Variability and performance of radiologic stricture parameters in Crohn's disease: a systematic review and meta-analysis
Source: eClinicalMedicine. 2025 Oct 8;89:103541. doi: 10.1016/j.eclinm.2025.103541 (PMC12538911; doi:10.1016/j.eclinm.2025.103541)
Supplement: Caption for Supplementary Material [file mmc2.docx]

**Caption for supplementary material**

**Supplementary materials**

The detailed primary search strategy for the systematic review.

**Supplementary PRISMA 2020 checklist**

RISMA checklist for reporting systematic reviews.

**Supplementary Table 1. Detailed search strategy for systematic review**

List of databases, search terms, and filters used.

**Supplementary Table 2. QUADAS-2 analysis of included studies.**

Abbreviations: ☺=Yes; ☹= No; ?= Unclear;

QUADAS-2 is structured so that 4 key domains are each rated in terms of the risk of bias and the concern regarding applicability to the research question (as defined above). Each key domain has a set of signaling questions to help reach the judgments regarding bias and applicability. 1. Was a consecutive or random sample of patients enrolled? 2. Was a case-control design avoided? 3. Did the study avoid inappropriate exclusions? 4. Were the index test results interpreted without knowledge of the results of the reference standard? 5. If a threshold was used, was it pre-specified? 6. Is the reference standard likely to correctly classify the target condition? 7. Were the reference standard results interpreted without knowledge of the results of the index test? 8. Was there an appropriate interval between index test(s) and reference standard? 9. Did all patients receive a reference standard? 10. Did patients receive the same reference standard? 11. Were all patients included in the analysis?

**Supplementary Table 3. Sensitivity analysis excluding studies at high risk of bias**

Values are pooled sensitivity and specificity (%) with I² heterogeneity index. Excluding studies judged at high risk of bias by QUADAS-2 produced minimal absolute differences, indicating robustness of the primary findings.

**Supplementary Table 4. Leave-one-out sensitivity analysis.**

Each study was sequentially omitted from the meta-analysis to assess its influence on pooled estimates. Values are pooled sensitivity and specificity (%) after exclusion, with delta values showing the absolute change from the overall model.

**Supplementary Table 5. Summary of Findings (GRADE) for diagnostic accuracy of US, MRE, and CTE in detecting small-bowel Crohn’s disease strictures.**

GRADE domains considered: risk of bias (QUADAS-2), inconsistency (I², overlap of CIs), indirectness (surgical cohorts; applicability to broader CD populations), imprecision (width of 95% CIs; total information size), publication bias (not formally assessed given small numbers).

Pooled estimates come from the bivariate random-effects model in the primary analysis; IUS includes SICUS where applicable.

Ratings reflect certainty within each modality vs histopathology; they do not rank modalities against each other.

Where heterogeneity and/or CIs were wide, this uncertainty is reflected in the GRADE rating and narrative.
